# Geometric morphometric analysis of the pronotum and elytron in stag beetles: insight into its diversity and evolution

**DOI:** 10.3897/zookeys.833.26164

**Published:** 2019-03-25

**Authors:** Mengna Zhang, Yongying Ruan, Xia Wan, Yijie Tong, Xingke Yang, Ming Bai

**Affiliations:** 1 Key Laboratory of Zoological Systematics and Evolution, Institute of Zoology, Chinese Academy of Sciences, Box 92, Beichen West Road, Chaoyang District, Beijing, 100101, China Institute of Zoology, Chinese Academy of Sciences Beijing China; 2 School of Applied Chemistry and Biological Technology, Shenzhen Polytechnic, Shenzhen, 518055, China School of Applied Chemistry and Biological Technology Shenzhen China; 3 Department of Ecology, School of Resources & Engineering, Anhui University, Hefei 230601, China Anhui University Hefei China

**Keywords:** Elytron, geometric morphometrics, morphological diversity, pronotum, species richness, stag beetle

## Abstract

Stag beetles (Coleoptera, Scarabaeoidea, Lucanidae) have received extensive attention from researchers in behavioral ecology and evolutionary biology. There have been no previous quantitative analyses, particularly using a geometric morphometric approach based on a large sample of data, to shed light on the morphological diversity and evolution of Lucanidae. Thoracic adaptation and ecological differentiation are intimately related, and the pronotum bears important muscles and supports the locomotion of prothoracic legs. The elytron is an autapomorphy of the Coleoptera. To reconstruct and visualize the patterns of evolutionary diversification and phylogenetic history of shape change, an ancestral groundplan can be reconstructed by mapping geometric morphometric data onto a phylogenetic tree. In this study, the morphologies of the pronotum and elytron in 1303 stag beetles (Lucanidae), including approximately 99.2% of all globally described species, were examined, thus revealing several aspects of morphological diversity and evolution. First, on the basis of geometric morphometric analysis, we found significant morphological differences in the pronotum or elytron between any two Lucanidae subfamilies. And we subsequently reconstructed the ancestral groundplans of the two structures in stag beetles and compared them with those of extant species (through cladistic and geometric morphometric methods). The ancestral groundplan of Lucanidae was found to be most similar to extant Nicagini in both the pronotum and elytron, according to Mahalanobis distances. Furthermore, we analyzed species richness and morphological diversity of stag beetles and the relationships between them and found that the two parameters were not always correlated. Aesalinae was found to be the most diverse subfamily in both the pronotum and elytron, despite its poor species richness, and the diversity of the pronotum or elytron was not superior in Lucaninae, despite its high species richness. Our study provides insights into the morphological variations and evolutionary history of the pronotum and elytron in four subfamilies of stag beetles, and it illuminates the relationship between morphological diversity and species richness. Intriguingly, our analysis indicates that morphological diversity and species richness are not always correlated. These findings may stimulate further studies in this field.

## Introduction

Stag beetles (Lucanidae) comprise more than 1300 described species, which are grouped into over 100 genera and exist in all zoogeographical regions except Antarctica (Fujita 2010; Kim and Farrell 2015). Owing to their sexual dimorphism, male polymorphism, and unique behaviors, stag beetles have received extensive attention from coleopterists and evolutionary biologists.

In recent years, multiple aspects of stag beetle morphology have been studied, and numerous evolutionary interpretations have been proposed. For instance, a study of the evolution of the Lucanidae has suggested that negative wing allometry may reflect a morphological cost of evolving oversized mandibles (Kawano 1997), and finite-element modeling has revealed force modulation of jaw adductors in stag beetles (Goyens et al. 2014). In addition, some studies have attempted to address evolutionary questions in specific subfamilies, such as Penichrolucaninae (Ratcliffe, 1984). Hosoya and Araya (2005, 2006) have inferred the phylogeny and evolution of Japanese stag beetles from morphological characters and 16S mtrRNA gene sequences, and the same team has investigated the phylogeny of the genus *Dorcus* and its allied genera by using allozyme or molecular data (Hosoya et al. 2002, 2003; Hosoya 2011). Furthermore, a new genus has been proposed, and a phylogenetic tree of Lucanidae based on two gene regions of ribosomal DNA has indicated the monophyly of four subfamilies (Paulsen 2013). Both morphological diversity and species richness are important in the study of diversity. The species richness of stag beetles has been revealed through various monographs (Fujita 2010; Maes and Pinratana 2003; Paulsen 2010), descriptions of new species (Okuda 2012b; Nguyen 2013) and reviews of certain taxa (Paulsen and Mondaca 2006; Huang and Chen 2012).

Among the morphological characters of stag beetles previous research was focused on the mandibles (Kawano 2003; Knell et al. 2004; Knight 2014), allometry (Kawano 2000; Tatsuta et al. 2001; Hardersen et al. 2011), sexual dimorphism or male polymorphism (Kawano 2006; Iguchi 2013), and genitalia (Tatsuta et al. 2001; Imura 2007). The pronotum and elytron have typically been used as indexes for body size (Tatsuta et al. 2004; Chiari et al. 2014), both of which contain important information about the evolution of the Lucanidae. Thoracic adaptation and ecological differentiation are intimately related and, differences in size, structure and function in the prothorax are readily perceived and correlated with physical demands of various environments (Hlavac 1972). As a part of the prothorax, the pronotum bears important muscles and supports the locomotion of the prothoracic legs (Evans 1977). In fact, the muscles in the prothorax of a stag beetle are hypertrophied to help raise the head while lifting opponents (Goyens et al. 2015). The elytron is an autapomorphy of the Coleoptera, which was being transformed into elytra in the Permian (Beutel and Leschen 2005; Ponomarenko 2004). These two traits are also correlated to mandibles in morphology. The mandibles of most males are highly developed, thus contributing to the morphological diversity of the stag beetles. The species with relatively large mandibles have proportionally enlarged prothorax and smaller wings, which may be developmental integration trade-offs generated by resource competition between characters (Kawano 1997; Okada and Miyatake 2009). As mandible shape has wide variation even within the same species between large and small males in stag beetles, this study focuses on the pronotum and elytron, which are relatively conservative within species and more feasible for a large sample size.

However, no quantitative analyses have been conducted, especially through a geometric morphometric approach (Bai et al. 2012, 2014b; Friedman 2010), to reveal the morphological diversity and evolution of Lucanidae on the basis of a large sample size. Additionally, the relationship remains unclear between the species richness and morphological diversity of the subfamilies of Lucanidae . To reconstruct and visualize the phylogenetic history of shape change, the phylogeny can be projected into the shape tangent space, and provides intuitive graphical displays that show, as far as it is possible to infer from the shape information of terminal taxa, how specific clades diversified and spread through the space of morphometric variables (Klingenberg and Marugán-Lobón 2013).

Three major aspects of pronotum and elytron morphology of stag beetles were investigated in this study. First, the morphological variations in the pronotum and elytron of 1303 stag beetles were analyzed through a geometric morphometric approach. Second, the ancestral groundplans of the pronotum and elytron of the subfamilies of Lucanidae were reconstructed, and the evolution of the two structures was inferred and discussed. Furthermore, the species richness and morphological diversity of four subfamilies were compared.

## Methods

### Taxa examined

This study analyzed 1447 species including 1303 lucanid species and 144 outgroup species. All four subfamilies (Aesalinae, Lampriminae, Lucaninae, and Syndesinae), all 105 lucanid genera, and 1303 lucanid species (approximately 99.2% of all described lucanid species) from around the world were included as the inner group, and the outgroups consisted of 4 Diphyllostomatidae species, 16 Hybosoridae species, 16 Geotrupidae (Geotrupinae+Bolboceratinae) species, 11 Passalidae (Passalinae+Aulacocyclinae) species, 6 Glaresidae species, 19 Ochodaeidae species, 43 Scarabaeidae species, 9 Trogidae species, 12 Silphidae species, and 8 Histeridae species. The measurements of all lucanid species and most outgroup species were based on published images (photographs or specimen drawings) (Dallwitz 1980; Dallwitz et al. 1993, 1995, 2000; Sakai and Nagai 1998; Bunalski 1999; Král 2001; Arnett et al. 2002; Ocampo 2006; Mondaca and Smith 2008; Schenk 2008; Nikolajev 2009; Bomans 2010; Fujita 2010; Imura 2010, 2011, 2012; Boilly 2011; Kobayashi and Matsumoto 2011; Okajima and Araya 2012; Okuda 2012a; Palestrini et al. 2012; Paulsen and Ocampo 2012; Ballerio 2013; Král et al. 2013; Paulsen 2013; Bezborodov and Koshkin 2014), and those of the Passalidae species were based on the specimens housed in the Institute of Zoology of the Chinese Academy of Sciences (Suppl. material 1: Table S1). In consideration of sexual dimorphism and male polymorphism in Lucanidae, images of the male specimens were selected in this study, as they are more accessible in publications, and medium-sized specimens were chosen if there were images of different body sizes available.

### Data analysis

**Figure 1.**
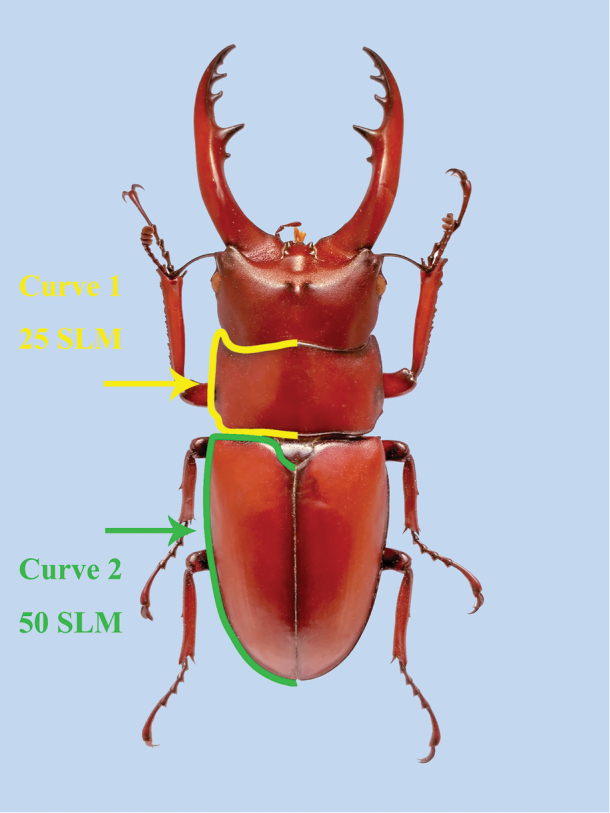
Description of the curves used in geometric morphometric analysis. The positions selected for the pronotum and elytron curves are represented by *Prosopocoilus* sp. in dorsal view. The curves were resampled in 25 or 50 semi-landmarks (SLM).

**Figure 2.**
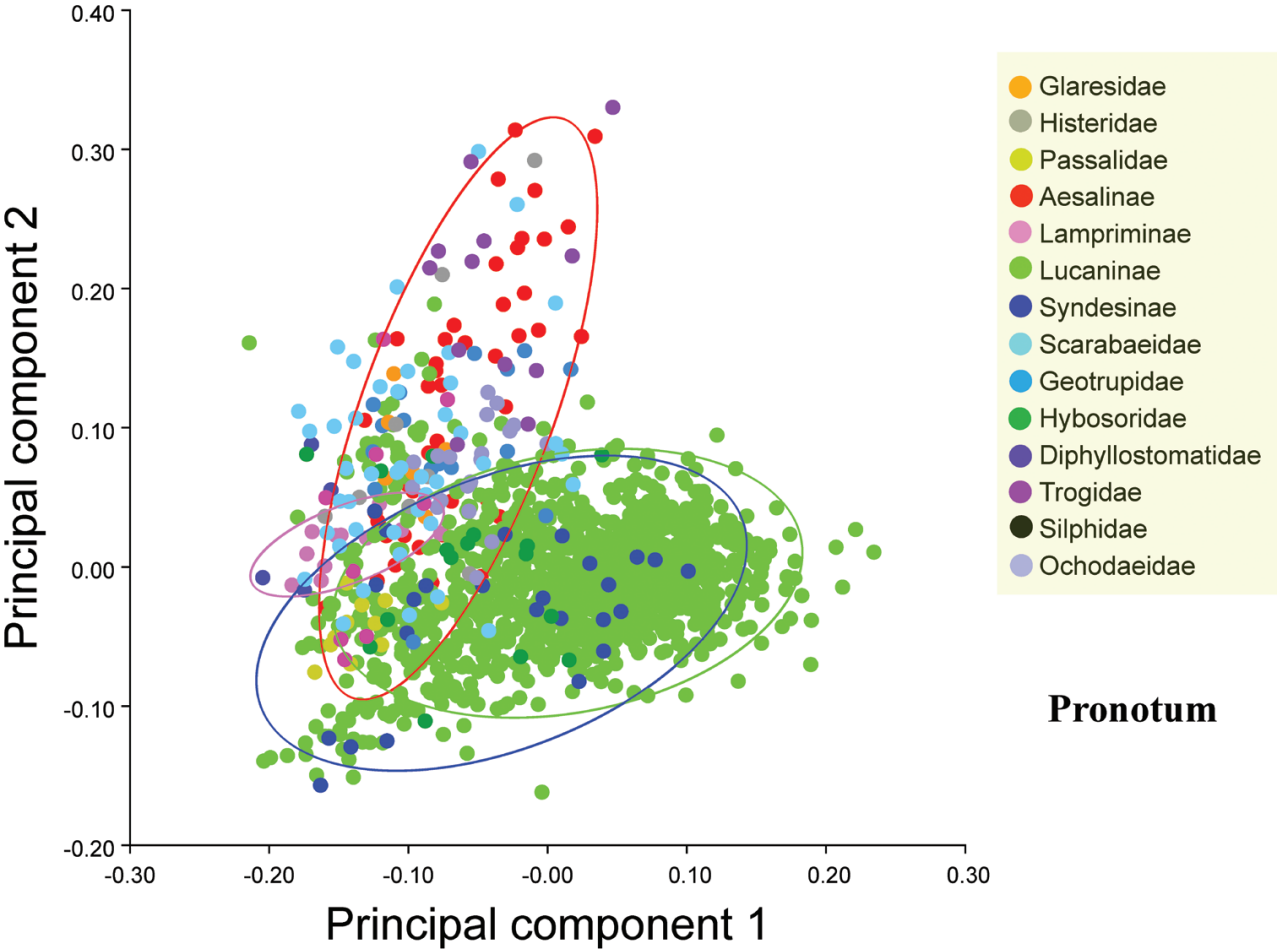
Differences in pronotum shape between outgroups and Lucanidae, on the basis of principal component analysis at the species level. The four circles are 90%-equal frequency ellipses of Lucanidae subfamilies.

**Figure 3.**
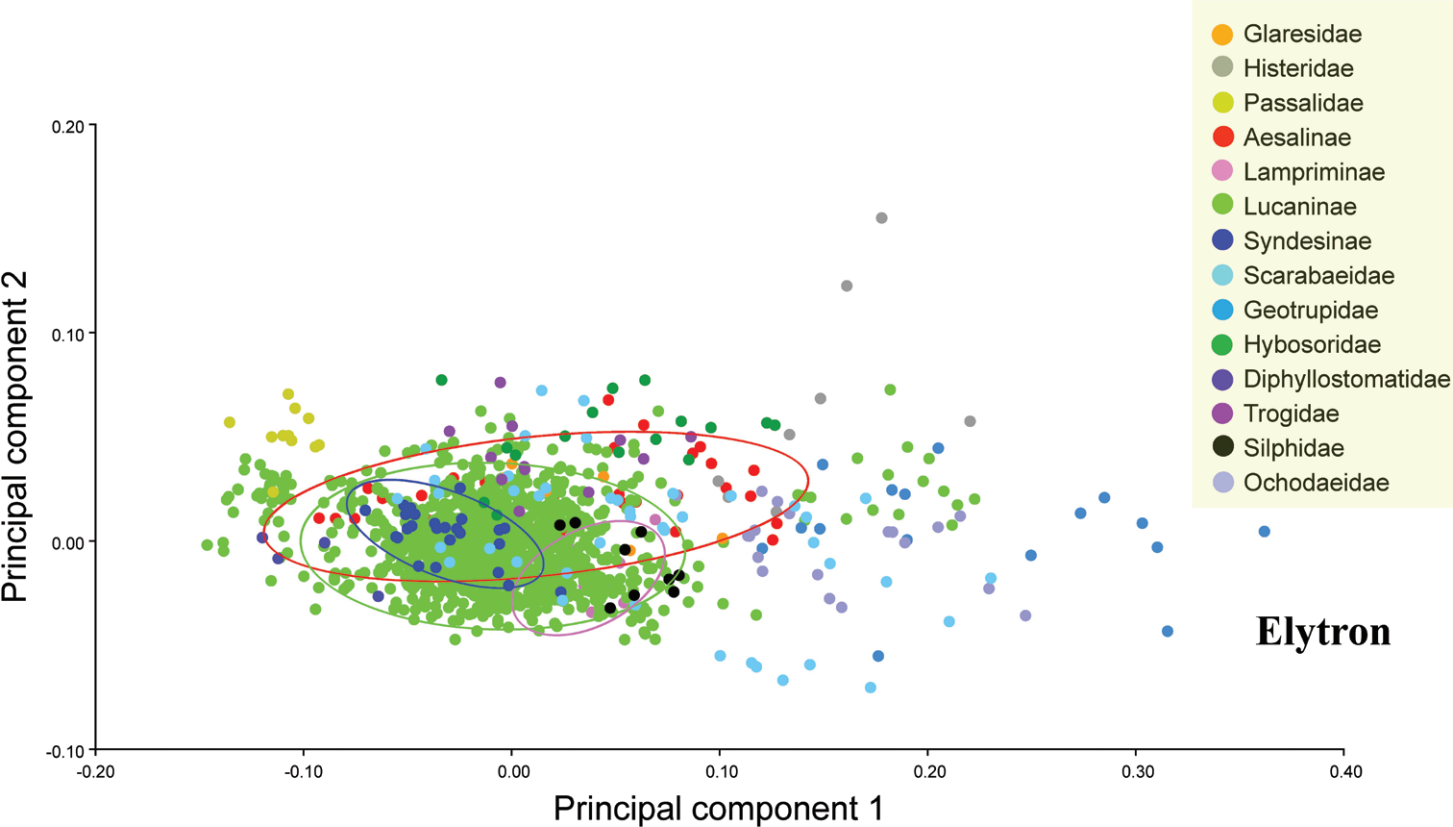
Differences in elytron shape between outgroups and Lucanidae, on the basis of principal component analysis at the species level. The four circles are 90%-equal frequency ellipses of Lucanidae subfamilies.

Geometric morphometric analysis of the variations in the pronotum and elytron were based on one curve for each structure (Fig. 1), and the curves for the pronotum or elytron were resampled by length after 25 and 50 semi-landmarks (SLM), respectively. The curves were digitized with TPS-DIG 2.05 (Rohlf 2006), and the data file was modified as .txt file to convert the semi-landmarks to landmarks (MacLeod 2017). The two lines with the curve number and point number were deleted, and the landmark number was replaced by the point number. This approach was used earlier by Bai et al. (2014a) and Li et al. (2016). The landmark configurations were scaled, translated, and rotated against the consensus configuration by using the Procrustes superimposition method (Bookstein 1991). The differences in the shapes and diversity indexes of the pronotum and elytron were inferred on the basis of principal component analysis (PCA) in MORPHOJ 1.06a (Klingenberg 2011) (Figs 2, 3, 8, 9, Table 1, Suppl. material 1: Table S10). The diversity index was quantified as Procrustes variance, which measures the dispersion of all observations around the mean shape of the respective taxa (Zelditch et al. 2004; Sherratt et al. 2014). The association of morphological diversity with species richness at genus-level was measured using Pearsons correlation coefficient, r in PAST 3.01 software (Sherratt et al. 2014; Hammer et al. 2001) (Suppl. material 1: Table S10, S11).

**Table 1.** Species richness and morphological diversity of the pronotum and elytron at the subfamily level.

Subfamily	Species number	Total variance	
		Pronotum	Elytron
Aesalinae	47	0.0158	0.0044
Lampriminae	11	0.0030	0.0007
Lucaninae	1220	0.0111	0.0025
Syndesinae	25	0.0123	0.0008

**Figure 4.**
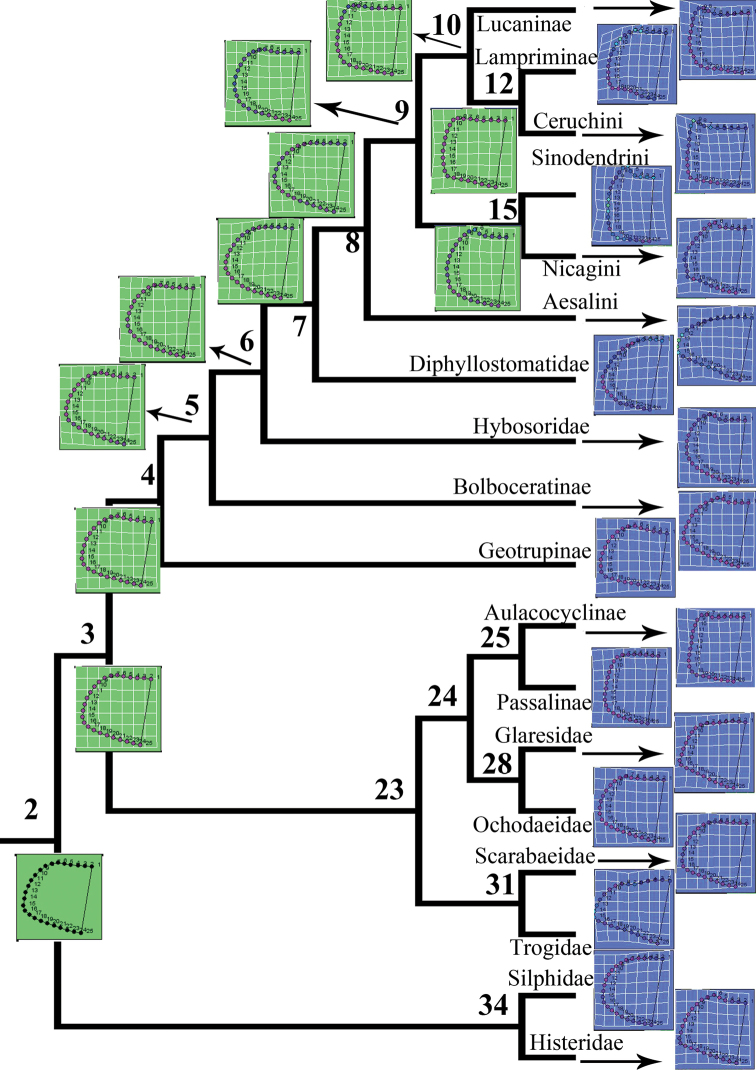
Reconstruction of ancestral groundplans of the pronotum in Lucanidae and the outgroups. The splines indicate deformation of the shapes relative to the reference configuration. The phylogenetic tree was summarized and reconstructed from earlier molecular results (Kim and Farrell 2015).

**Figure 5.**
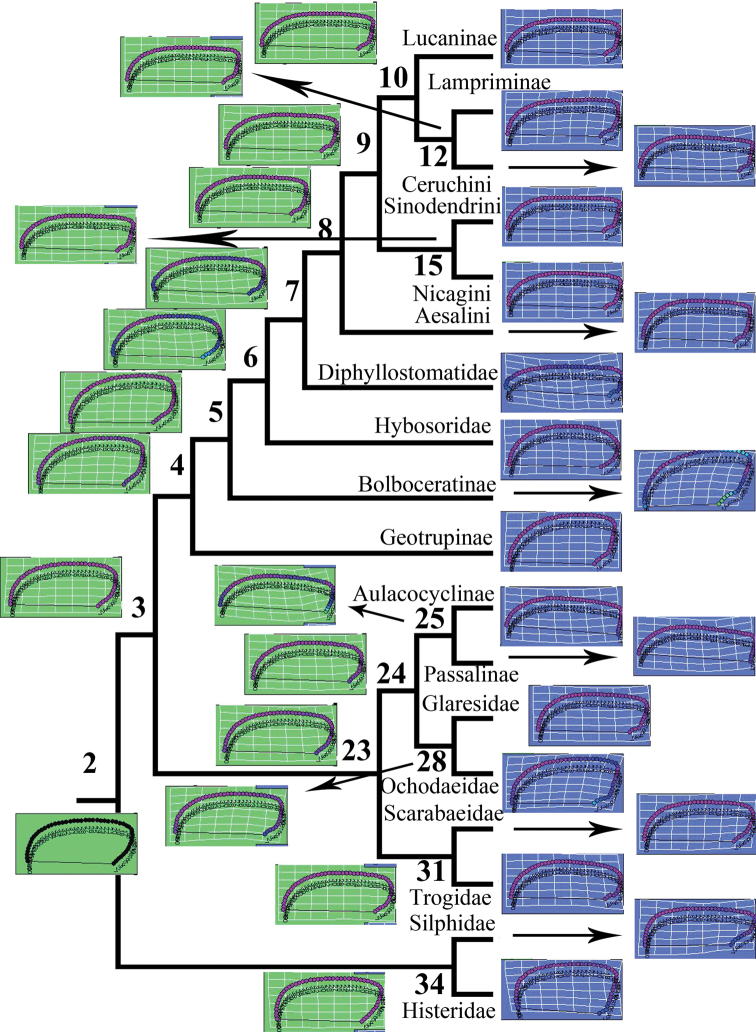
Reconstruction of ancestral groundplans of the elytron in Lucanidae and the outgroups. The splines indicate the deformation of the shapes relative to the reference configuration. The phylogenetic tree was summarized and reconstructed from earlier molecular results (Kim and Farrell 2015).

A phylogenetic tree was visualized in MESQUITE 2.72 (Maddison and Maddison 2011) on the basis of earlier molecular analysis (Kim and Farrell 2015), and the aligned landmark data were entered into MESQUITE 2.72 as a continuous matrix and linked to the tree (Figs 4, 5). Because the branch lengths (Grafen 1989) were missing, we followed the evaluation proposed by Klingenberg and Marugán-Lobón (Klingenberg and Marugán-Lobón 2013) and assigned an equal length to all branches (i.e., an evolutionary model with the same expected amount of morphological change on every branch was assumed).

The ancestral groundplans of the Lucanidae pronotum and elytron were reconstructed by combining the landmark data with the phylogenetic tree, and the ancestral groundplans of all nodes were reconstructed by using the trace-all-characters and/or landmark-drawing modules of the RHETENOR package in MESQUITE. The ancestral states of all nodes were calculated and exported, and the data computed for the nodes were integrated with the original landmark data for the two characteristics from the 1303 stag beetles in EXCEL and NTSYS-PC (Rohlf 2007), respectively. The thin-plate splines showing the deformation of the landmarks compared with the original computed by MESQUITE were mapped onto the phylogenetic tree (Figs 4, 5).

**Figure 6.**
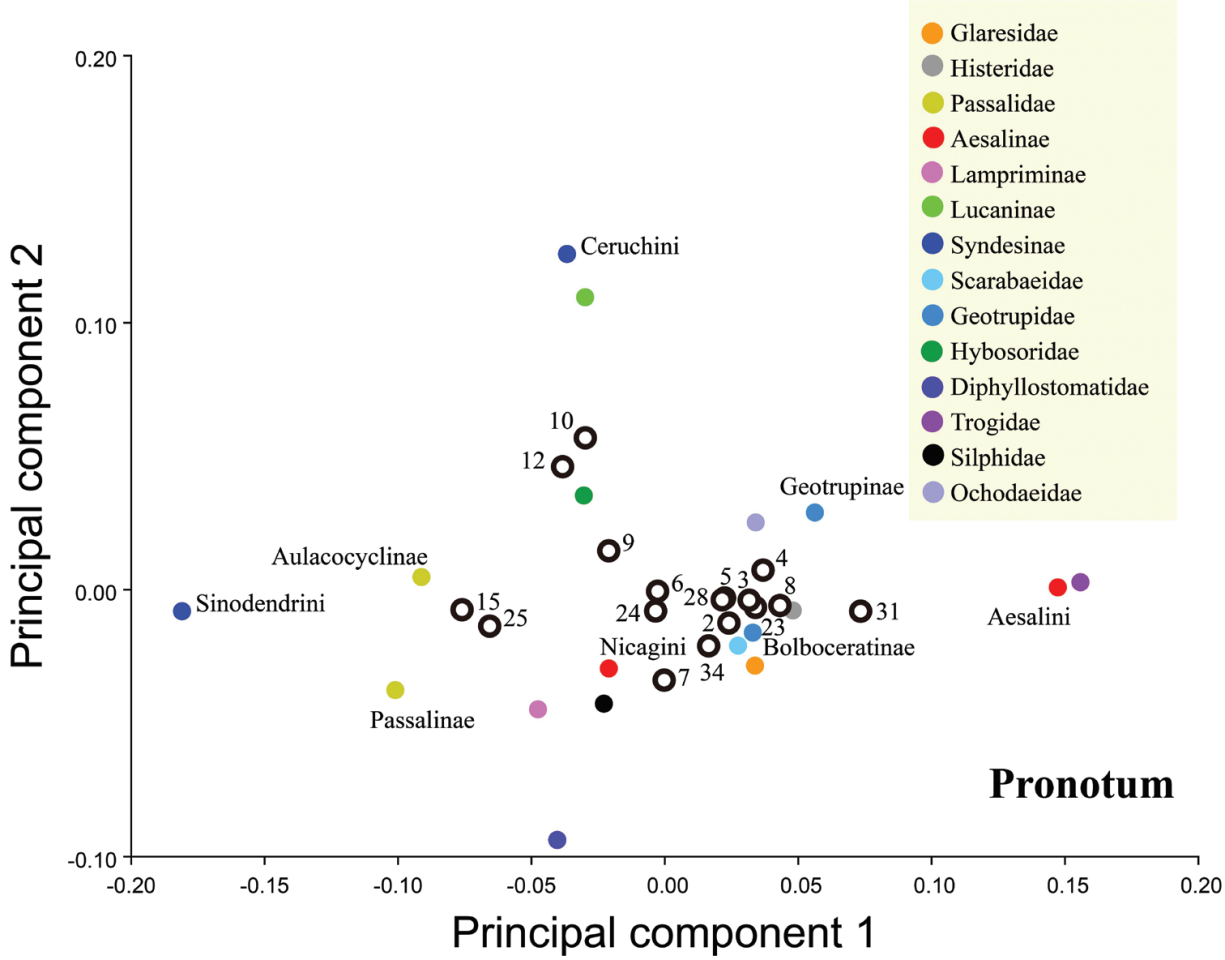
Differences in pronotum shape among each branch and ancestor, on the basis of principal component analysis. Empty dots indicate the number of the node on the phylogenetic tree; solid dots indicate the average shape of the extant subfamily/tribe of each branch.

**Figure 7.**
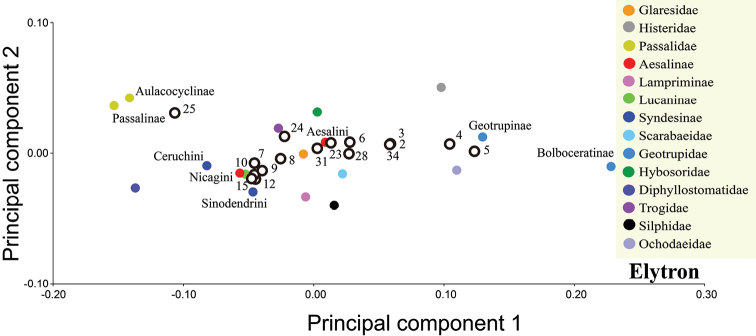
Differences in elytron shape among each branch and ancestor, on the basis of principal component analysis. Empty dots indicate the number of the node on the phylogenetic tree; solid dots indicate the average shape of the extant subfamily/tribe of each branch.

In this case, the differences in the shapes of the pronotum and elytron among extant and extinct Lucanidae were inferred on the basis of PCA in MORPHOJ 1.06a and PAST 3.01 (Figs 6, 7). The canonical variate analysis (CVA) and discriminant function analysis (DFA) of the landmark data were based on MORPHOJ 1.06a (Suppl. material 1: Tables S2–S9, S12, S13).

## Results

### Comparison of pronotum/elytron morphology among lucanid subfamilies

The first two principal components of the pronotum and elytron from all 1447 species accounted for 77.37% and 88.40% of the variation among the species, respectively. The first two principal components were plotted to indicate variation along the two axes, which provided 90% equal frequency ellipses containing approximately 90% of the data points of each group (Figs 2, 3). The pronotum morphologies of the outgroups were mostly within the entire morphological variation of the Lucanidae (Fig. 2).

All the *p*-values obtained from the permutation tests (10000 permutation rounds) for both Mahalanobis distances and Procrustes distances between any two Lucanidae subfamilies were less than 0.05 for both the pronotum and elytron. Most of the *p*-values for the pronotum and elytron Mahalanobis or Procrustes distances between the Lucanidae subfamilies and outgroups were less than 0.05, except for some of the distances between the Aesalinae or Lampriminae and the outgroups.

There were significant differences in both the pronotum and elytron between any two of the Lucanidae subfamilies (Suppl. material 1: Table S3, S5, S7, S9). For the pronotum, there was a significant difference between Lampriminae, Lucaninae, or Syndesinae and the outgroups; the Lucanidae and the outgroups partly overlapped, because the pronotum morphology of Aesalinae could not be distinguished from that of Geotrupidae, Glaresidae, Ochodaeidae, and Histeridae (Suppl. material 1: Table S3, S5). However, the differences in the pronotum among these four pairs were not equivalent. On the basis of the Procrustes distance, a measure of the absolute magnitude of the deviation in shape that indicates the extent of the differences between the average group shapes, the differences in the pronotum between Aesalinae and Geotrupidae, Glaresidae, Ochodaeidae, and Histeridae were 0.0512, 0.0637, 0.0488 and 0.0659, respectively (Table S4). For the elytron, there was a significant difference between Lucaninae or Syndesinae and the outgroups, but the morphology of Aesalinae and Lampriminae could not be distinguished from the morphologies of Glaresidae, Scarabaeidae, or Trogidae (Table S7, 9). There was greater overlap in the pronotum morphology of the Lucanidae subfamilies and outgroups than the elytron morphology (Suppl. material 1: Table S2, S6).

### Ancestral reconstruction of the pronotum and elytron

In a comparison of the ancestor of Lucanidae and all outgroups (node 2 in Fig. 4), the anterior angle of the pronotum of the ancestor of Lucanidae (node 8 in Fig. 4) is more obtuse (landmarks 4–12 at the splines in Fig. 4). It is shown in Figure 6 that a clear divergence between the two lineages of Lucanidae, the Aesalini lineage and lineage I (node 9 in Fig. 4), is primarily in the direction of the first principal component. Moreover, within lineage I, there is continued diversification in the direction of the first principal component (the horizontal direction in Fig. 6). The pronotum morphology of Lampriminae and Ceruchini showed clear changes in different directions, almost in the inverse direction of the second principal component (the vertical direction in Fig. 6), particularly in the anterior angle (landmarks 4–12 at the splines in Fig. 4).

The elytron of the ancestor of Lucanidae (node 8 in Fig. 5) is more slender than that of the outgroups (node 2 in Fig. 5) (the ratios of the length to the greatest width are 1.571 and 1.235, respectively) and narrower at the end (landmarks 35–50 at the splines in Fig. 5), but it is much wider than that of Diphyllostomatidae (the ratios of the length to the greatest width are 1.571 and 2.170, respectively). The elytron of Aesalini is wider than in lineage I (node 9 in Fig. 5) (the ratios of the length to the greatest width are 1.313 and 1.571, respectively), thus illustrating the major differences in the elytron between the two Lucanidae lineages. The humeral angle is narrower in the ancestor of Sinodendrini and Nicagini (node 15 in Fig. 5) than in the ancestor of Lucaninae, Lampriminae and Ceruchini (node 10 in Fig. 5) (landmarks 10–18 at the splines in Fig. 5).

### Species richness and pronotum and elytron morphology

In terms of the species richness, Lucaninae is the largest subfamily of Lucanidae, comprising more than 90% (1220) of the species of stag beetles, followed by Aesalinae, Syndesinae, and Lampriminae, which have fewer than 50 species each (47, 25, 11). However, morphological diversity of the pronotum and elytra shape does not correspond with species richness (Table 1). Aesalinae, with fairly low species richness, was found to be the most diverse subfamily in both pronotum and elytron morphology, whereas Lucaninae exhibited low morphological diversity (both in the pronotum and elytron). This is an unexpected result considering its extremely high species richness.

In 73 genera (all Lucanidae genera with more than one species), similarly to the subfamily data, the morphological diversity of neither the pronotum nor the elytron was consistent with species richness. There is no significant correlation between morphological diversity and species richness in pronotum at genus-level (Procrustes variance *r* = 0.15, *P* =0.21), nor a relationship between morphological diversity and species richness in elytron at genus-level (Procrustes variance *r* = 0.11, *P* = 0.33). The total morphological variances in extremely species-rich genera, such as *Aegus*, *Dorcus*, *Lucanus*, and *Prosopocoilus*, were not predominant.

## Discussion

### Evolution of the pronotum and elytron in Lucanidae

According to the Mahalanobis distances from the DFA (Suppl. material 1: Table S12, S13), all lucanid ancestors (nodes 8–10, 12, and 15) most resemble Nicagini in their pronotum and elytron morphology. Procrustes distances indicated that the pronotum of the ancestor of Lucanidae (node 8) is closest to that of Scarabaeidae, and the elytron is closest to that of Glaresidae. The common ancestor of Lucaninae, Lampriminae, Syndesinae, and Nicagini (node 9) most resembles Hybosoridae in the pronotum and Lucaninae in the elytron, and the ancestor of Lampriminae and Ceruchini (node 12) most resembles Hybosoridae in the pronotum and Sinodendrini in the elytron. The ancestor of Lucaninae, Lampriminae, and Ceruchini (node 10) is most similar to Lucaninae in both the pronotum and elytron, and as shown by both Mahalanobis and Procrustes distances, the ancestor of Sinodendrini and Nicagini (node 15) most resembles Nicagini in the pronotum as well as the elytron.

The ancestral pronotum and elytra were reconstructed, which could be combined with fossil materials to uncover the ancestors’ habitat as well as evolution procedure. There is still a lack of sufficient data as well as studies of the functional morphology of the pronotum and the elytron in Lucanidae, but the functional morphology of other insect clades may allow for certain interpretations. Broad pronotum and elytra of stag beetles may provide advantages during locomotion and hunting prey, like ground beetles (Evans 1977; Forsythe 1981). The diversity of pronotum may reflect occupancy of diverse habitats and niches, as in the case of grylloblattids (Bai et al. 2010).

**Figure 8.**
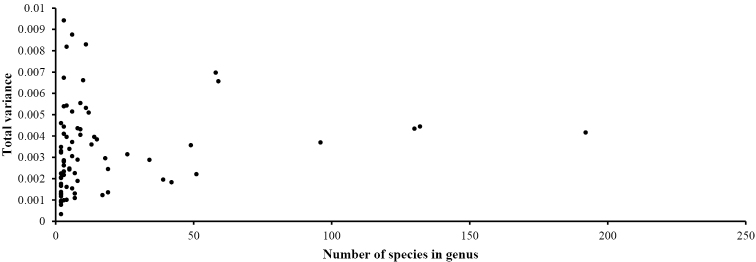
Species richness and morphological diversity of the pronotum at the genus level.

### The inconsistency between morphological diversity and species richness

Morphological and taxonomic diversity provide insight into the expansion and contraction of major taxa, and the nature of the relationship between these two aspects of diversity has important implications in evolutionary mechanisms (Foote 1993). Lampriminae has the lowest pronotum and elytron diversity as well as species richness, but species richness and morphological diversity do not always vary consistently in Lucanidae subfamilies. Despite having many more species than the other three subfamilies combined, the subfamily Lucaninae has little morphological diversity. Furthermore, the results from the genus-level data reveal the same pattern in which some of the largest genera are not as diverse as the small groups in terms of either pronotum or elytron morphology.

The theory that species richness generates a variety of forms has been tested and supported in various studies (Williams and Humphries 1996; Roy and Foote 1997; Bell and Barnes 2001), thus suggesting that the relationship between morphological and species diversity should be monotonic or at least positive.

In contrast, numerous studies have indicated that richness and morphology do not always follow a common trend. Foote (1993) has found that morphological variety and taxonomic richness often increase together during the initial diversification of a clade, but two major patterns have been observed as clades decline. In Blastoidea, Trilobita, Libristoma, and Asaphina, morphological diversity continued to increase even in the face of striking decreases in taxonomic richness, but in Phacopida, Scutelluina, and to some extent in Proetida, morphological diversity decreased along with taxonomic diversity. Roy et al. (2001), using data from a large group of IndoPacific gastropods (family Strombidae), have shown that the species richness of a region is a poor predictor of morphological diversity. Areas with only a few species may harbor an impressive array of morphologies, and in contrast, morphological diversity in the most species-rich regions is no higher than that in regions with half the taxonomic diversity. Bell and Barnes (2002) have found no significant correlation between species richness and morphological diversity in cave or boulder habitats, although these variables are significantly correlated in coral reef and soft substratum habitats. In another scenario (Mohedano-Navarrete et al. 2008), the morphological diversity and species richness of *Porites* corals has been found to vary independently; some regions with few species had remarkably high morphological diversity, including peripheral areas such as Polynesia and East Africa. In a study of 107 families of passerine birds, morphological space was weakly related to the number of species in a family, i.e., the higher species richness of the order Passeriformes in the tropics compared with temperate regions was not matched by increased morphological diversity (Ricklefs 2012).

**Figure 9.**
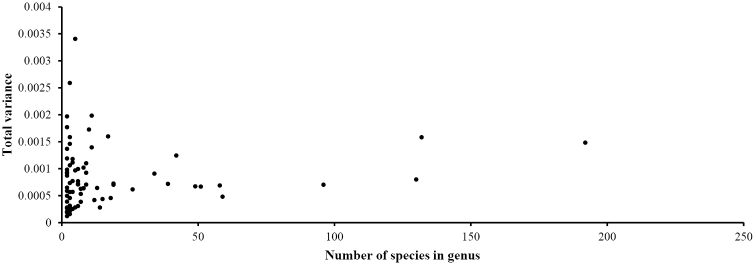
Species richness and morphological diversity of the elytron at the genus level.

## Conclusion

Our results showed significant differences in both pronotum and elytron morphology between any two lucanid subfamilies; in other words, the four subfamilies could be statistically separated and determined based on the two characters. On the basis of cladistic and geometric morphometric methods, the ancestral groundplans of the pronotum and elytron of extant Lucanidae were reconstructed and compared with those of extant species. The ancestor of Lucanidae is most similar to extant Nicagini in both pronotum and elytron morphology, according to Mahalanobis distances, but Procrustes distances indicated that the pronotum of the ancestor of Lucanidae is most similar to that of extant Scarabaeidae and that the elytron is most similar to that of extant Glaresidae. On the basis of a comparison of the four subfamilies as well as an analysis of Lucanidae genera, species richness and morphological diversity do not generally correlate. Lampriminae has the poorest morphological diversity in the pronotum and elytron as well as the poorest species richness, whereas Aesalinae is the most diverse subfamily with respect to both the pronotum and elytron, despite its small number of species.

However, the analyses are relatively limited, as there is morphological variation within species especially in male polymorphic stag beetles, and only one image per species was sampled during the procedure. In addition, as our results were limited to the morphological characters of the pronotum and elytron from the dorsal view, the investigation of more traits and groups should improve the understanding of the relationship between morphological diversity and species richness in beetles.

## Supplementary materials

Supplementary material 1 Geometric morphometric data Data type: morphometric data Explanation note: Tables S1–S12. This dataset is made available under the Open Database License (http://opendatacommons.org/licenses/odbl/1.0/). The Open Database License (ODbL) is a license agreement intended to allow users to freely share, modify, and use this Dataset while maintaining this same freedom for others, provided that the original source and author(s) are credited.Mengna Zhang, Yongying Ruan, Xia Wan, Yijie Tong, Xingke Yang, Ming Bai
